# Reliable Indoor Pseudolite Positioning Based on a Robust Estimation and Partial Ambiguity Resolution Method

**DOI:** 10.3390/s19173692

**Published:** 2019-08-25

**Authors:** Xin Li, Guanwen Huang, Peng Zhang, Qin Zhang

**Affiliations:** 1 College of Geology Engineering and Geomantic, Chang’an University, Xi’an 710054, China; 2 School of Geodesy and Geomatics, Wuhan University, Wuhan 430079, China

**Keywords:** indoor pseudolite (PL), standard unscented kalman filter (SUKF), robust unscented kalman filter (RUKF), partial ambiguity resolution (PAR), differential PL (DPL), real-time kinematic (RTK) positioning

## Abstract

The unscented Kalman filter (UKF) can effectively reduce the linearized model error and the dependence on initial coordinate values for indoor pseudolite (PL) positioning unlike the extended Kalman filter (EKF). However, PL observations are prone to various abnormalities because the indoor environment is usually complex. Standard UKF (SUKF) lacks resistance to frequent abnormal observations. This inadequacy brings difficulty in guaranteeing the accuracy and reliability of indoor PL positioning, especially for phase-based high-precision positioning. In this type of positioning, the ambiguity resolution (AR) will be difficult to achieve in the presence of abnormal observations. In this study, a robust UKF (RUKF) and partial AR (PAR) algorithm are introduced and applied in indoor PL positioning. First, the UKF is used for parameter estimation. Then, the anomaly recognition statistics and optimal ambiguity subset of PAR are constructed on the basis of the posterior residuals. The IGGIII scheme is adopted to weaken the influence of abnormal observation, and the PAR strategy is conducted in case of failure of the conventional PL-AR. The superiority of our proposed algorithm is validated using the measured indoor PL data for code-based differential PL (DPL) and phase-based real-time kinematic (RTK) positioning modes. Numerical results indicate that the positioning accuracy of RUKF-based indoor DPL is higher with a decimeter-level improvement compared that of the SUKF, especially in the presence of large gross errors. In terms of high-precision RTK positioning, RUKF can correctly identify centimeter-level anomalous observations and obtain a corresponding positioning accuracy improvement compared with the SUKF. When relatively large gross errors exist, the conventional method cannot easily realize PL-AR. By contrast, the combination of RUKF and the PAR algorithm can achieve PL-AR for the selected ambiguity subset successfully and can improve the positioning accuracy and reliability significantly. In summary, our proposed algorithm has certain resistance ability for abnormal observations. The indoor PL positioning of this algorithm outperforms that of the conventional method. Thus, the algorithm has some practical application value, especially for kinematic positioning.

## 1. Introduction

Pseudo-Satellite or Pseudolite, abbreviated as PL, is a transmitter deployed on the ground to transmit some kind of positioning signal, which usually transmits signals similar to navigation satellite system (GNSS) [1,2,3]. PL can be used as a supplement or enhancement for GNSS, especially in complex environments [4,5,6]. The PL system has been proven to have an independent positioning capability similar to GNSS and can be a representative technology of indoor high-precision positioning [7,8,9,10]. The increase in the demand for high-precision indoor positioning and the development of PL hardware technology and user receiver have increased the research attention on the indoor PL positioning technology. In fact, for indoor positioning, many technologies have been developed for various applications, such as Wi-Fi, Radio Frequency Identification (RFID), ultra-wideband (UWB), etc. [11,12,13]. Compared with most of the existing methods, the indoor PL positioning technology is relatively high-cost and has a high positioning precision; however, the corresponding data processing is more complex.

The code and carrier phase observations of an indoor PL system contain relatively fewer error sources than GNSS [14]. In general, tropospheric and ionospheric errors do not exist in indoor environments. The clocks of PLs and receivers are considerably more low cost than those used by GNSS. Thus, the clock error is the predominant error source of PL observations. The PL system represented by LOCATA has solved the clock difference problem by using complex clock synchronization technology [15]. However, this problem is still difficult for most of the current relatively low-cost indoor PL systems. We can draw lessons from the relative positioning model in GNSS; the double-differenced (DD) observations can effectively eliminate or weaken most error sources, such as the clock error and antenna phase center deviation, and can thus greatly simplify the difficulty in PL data processing [16]. The code-based differential PL (DPL) and phase-based real-time kinematic (RTK) positioning models are usually adopted for indoor PL positioning depending on the type of observation [17]. 

PL antennas are usually fixed installations. This condition changes the geometric structure between the carrier and PLs slowly, which is not conducive to indoor PL positioning, especially for static observation. In this observation, the multi-epoch equation has a strong correlation. Thus, static multi-epoch cumulative observation is unsuitable for estimating the coordinate and ambiguity parameters [18]. To solve this problem, the known point initialization (KPI) can be adopted; the receiver in this method is placed at a fixed point with known coordinates to obtain the float ambiguity solution [19]. In general, KPI requires the known point with a relatively accurate priori coordinate information, which may bring some inconvenience in practical application. Although some scholars have used algorithms to overcome the problem of KPI [17,18], the reliability and stability in practical application remain difficult to guarantee. Therefore, KPI without the assistance of other sensors is still the commonly used approach for high-precision indoor PL positioning among the existing low-cost PL systems. For high-precision PL positioning, ambiguity resolution (AR) is important and intractable. In this step, the familiar LAMBDA method [20,21] commonly used in GNSS filed can be as an alternative approach for indoor PL system, which has been demonstrated in some previous studies [22].

When conducting parameter estimation for indoor PL positioning, the extended Kalman filter (EKF) [23,24] is usually adopted, while EKF inevitably brings linearized errors in a small space [25,26]. Li et al. [27] adopted the unscented Kalman filter (UKF) to solve this problem, which is a nonlinear parameter estimation method and with a better performance for indoor PL positioning. UKF [28] is based on UT transform, which abandons the traditional linearization procedure for nonlinear functions. The UKF algorithm is based on the framework of Kalman, and the unscented (UT) transform is used to deal with the nonlinear transfer of mean and covariance. UKF approximates the probability density distribution of the nonlinear function. It uses a series of definite samples to approximate the posterior probability density of the state, instead of approximating the nonlinear function. The UKF does not need to calculate Jacobian matrix, and avoids linearization, which usually neglects higher order terms; thus, the calculation accuracy of UKF is higher than that of EKF [29,30]. UKF is widely used in many fields such as navigation, target tracking, signal processing and neural network learning. Li et al. [27] had proved the superiority of UKF for indoor PL positioning, mainly due to its higher positioning accuracy and lower dependence on initial values compared with additional EKF.

In summary, adopting UKF based on the relative model and KPI method for parameter estimation and LAMBDA for PL-AR can achieve a decimeter-level positioning precision in DPL and a centimeter-level positioning precision in RTK under a good observation environment [27]. However, the indoor observation environment is often complex in practical application. The multipath effect of code observation and other interference factors may exist, and frequent observation abnormalities will negatively affect the accuracy and reliability of indoor PL positioning. Standard UKF (SUKF) lacks resistance to various abnormal situations [31,32]. Some scholars have put forward robust estimation theory to resist the influence of abnormal errors in measurement information [33,34]. The essence is to control the gross errors by constructing equivalent weights for weakening the influence of abnormal errors on the solution. This method reduces the contribution of anomalous observations to parameter estimation and can yield a relatively reliable result [35,36]. The recursive form of robust UKF (RUKF) is consistent with that of SUKF. The only difference between them is that the variance matrix of measurement noise in the observation model is replaced by the equivalent variance matrix [32,37]. Several familiar equivalent weight functions, such as IGG (I–III), Andrew, Tukey, and Huber methods, are usually adopted to calculate the equivalent weight matrix [38,39]. For indoor PL high-precision RTK, the RUKF will weaken the abnormal effects and improve the accuracy of float ambiguity solution when observation anomalies exist. However, all ambiguities cannot be guaranteed to be fixed successfully, especially under the influence of large gross errors. A partial AR (PAR) strategy is often adopted in the GNSS field to solve the aforementioned problem [40,41]. That is, if all ambiguity elements cannot be fixed reliably, then we can consider partial ambiguity elements that can be easily fixed. The PAR strategy is used to select the optimal ambiguity subset from all ambiguity elements and make a de-correlation for them. Then, the LAMBDA method is utilized to search the integer ambiguity solution for the selected ambiguity subset. Finally, the remaining ambiguity subset and coordinate solution are updated. In the GNSS field, the signal-to-noise ratio (SNR), elevation angle, posterior observation residual, the estimated variance matrix of float ambiguity, and the bootstrapping AR success rate or ratio value are usually used as the alternative information for selecting the optimal ambiguity subset [42]. Some scholars have successfully applied the robust Kalman filter to GNSS positioning and integrated navigation [43,44,45]. Most previous studies on PL positioning have focused on the outdoor simulated code-based meter-level positioning system and are thus not convincing [46,47,48]. Nearly no works have been performed on the RUKF-based indoor PL positioning, especially for the measured system and PAR-based high-precision RTK positioning.

In this study, we propose a reliable indoor PL positioning method based on the RUKF and PAR algorithm. First, the UKF is used for parameter estimation on the basis of posterior residuals. The standardized residues and anomaly recognition statistics are calculated. Then, the IGGIII scheme is used to construct the equivalent weight matrix for weakening the influence of abnormal observation. We further combine the RUKF with PAR strategy to obtain reliable PL positioning results with certain robustness by the anomaly recognition statistics information and select an ambiguity subset for PL-AR in the presence of large gross errors in the carrier phase observation. In Section 2, the specific method of our proposed method is provided. In Section 3, the superiority of the proposed method is verified using static and kinematic data for indoor DPL and RTK positioning. In Section 4, some conclusions are provided.

## 2. Methods

### 2.1. Indoor PL Positioning Model 

For a single-frequency PL system, suppose that n+1 PLs are observed synchronously on one epoch, 2n DD observation equations can be formed, namely, n DD code (p) and n DD carrier phase (ϕ) observation equations, which can be described as:

(1) $$\begin{array}{l} \varphi_{qr}^{ls}=\rho_{qr}^{ls}+\lambda N_{qr}^{ls}+\varepsilon_{qr}^{ls}\\ p_{qr}^{ls}=\rho_{qr}^{ls}+e_{qr}^{ls} \end{array}$$

where the subscripts q and r denote referenced and rover stations, respectively; the superscripts l and s denote referenced and non-referenced PLs, respectively; $N_{qr}^{ls}$ denotes the PL DD ambiguity; the $\varepsilon_{qr}^{ls}$ denotes DD carrier phase observation noise, and the $e_{qr}^{ls}$ is DD code observation noise; For the indoor PL DD observations, the clock errors and phase center offset can be eliminated; $\rho_{qr}^{ls}=(\rho_{r}^{s}-\rho_{r}^{l})-(\rho_{q}^{s}-\rho_{q}^{l})$ is the DD geometric distance between the receiver and PL. The mathematical model for the indoor PL relative positioning can be described as follows:

(2) $$y=E\left[\begin{array}{c} \phi \\ p \end{array}\right]=\left[\begin{array}{cc} \Gamma & \Lambda \\ \Gamma & 0 \end{array}\right]\left[\begin{array}{c} a\\ b \end{array}\right]D\left[\begin{array}{c} \phi \\ p \end{array}\right]=\left[\begin{array}{cc} Q_{\phi \phi } & 0\\ 0 & Q_{pp} \end{array}\right]$$

where $E\left[{}_{•}\right]$ and $D\left[{}_{•}\right]$ denote the expectation and dispersion operators, respectively; a is the real-valued baseline vector; and b is the integer ambiguity vector. Γ denotes the design matrix that corresponds to a and Λ denotes the design matrix that corresponds to b. $Q_{\phi \phi }$ denotes the covariance matrix that corresponds to the PL DD phase observation vectors, and $Q_{pp}$ denotes the covariance matrix that corresponds to the PL DD code observation vectors. The stochastic model for PL positioning generally uses an elevation-dependent weight model. The corresponding observation noise of the code and carrier phase can be pre-given approximate to GPS L1, and the noise can be obtained by prior modeling for PL observations.

For the phase-based RTK model, if n+1 PLs are synchronously observable, then n DD observation equation can be formed, and the contribution of code observation to high-precision RTK is generally ignored due to its poor accuracy. Thus, n+3 parameters need to be estimated (n PL DD ambiguity and 3 coordinate parameters). Evidently, the number of single-epoch observations is less than the number of parameters to be estimated. For a PL system, multi-epoch static observation cannot be conducted to increase the number of observations due to strong correlation of static observations on successive epochs. Therefore, the coordinate should be regarded as an a priori value when PL ambiguity is solved in RTK. Thus, the KPI method is usually adopted for current PL systems.

### 2.2. Robust UKF

For a nonlinear system, the state and observation equation can be represented by:

(3) $$\begin{array}{l} x_{k}=f(x_{k-1})+w_{k}\\ y_{k}=h(x_{k})+v_{k} \end{array}$$

where k is the discrete time; $x_{k}$ is the state parameter to be estimated, in this study, it contains the coordinates and PL ambiguity parameters; $y_{k}$ is the measurement containing PL DD code and carrier phase observations; f(⋅) denotes the state function and is an identity matrix in this study; h(⋅) is a nonlinear observation function which can be seen as Equation (1); $w_{k}$ and $v_{k}$ are the corresponding Gaussian white noises which meet the following equation:

(4) $$\begin{array}{l} E(w_{i})=0,E(w_{i}w_{j}^{T})=\delta_{ij}\cdot Q_{i}\\ E(v_{i})=0,E(v_{i}v_{j}^{T})=\delta_{ij}\cdot R_{i}\\ E(w_{i}v_{j}^{T})=0 \end{array}$$

where $\delta_{ij}$ is the Kronecker−δ function; Q denotes the variance matrices of the process noise, R denotes the variance matrices of the measurement noise.

The recursive form of RUKF is similar to that of SUKF. Their only difference is that the variance–covariance (VC) matrix of the measurement noise of the observation model is replaced by the equivalent variance matrix.

In this study, the calculation steps of RUKF algorithm are summarized as follows:

Step 1: Initialization of the state parameters.

(5) $$\begin{array}{l} {\hat{x}}_{0}=E(x_{0})\\ P_{0}=E[(x_{0}-{\hat{x}}_{0})(x_{0}-{\hat{x}}_{0}){}^{T}] \end{array}$$

where ${\hat{x}}_{0}$ denotes the initial state parameters including the 3-D coordinate and PL DD ambiguity, and $P_{0}$ denotes the corresponding covariance matrices of ${\hat{x}}_{0}$.

Step 2: Generation of sigma points.

In this step, the sampling strategy based on Cholesky decomposition is usually utilized, for the n-dimensional variables $x_{k-1}$ (with the mean and variance of ${\hat{x}}_{k-1}$ and $P_{k-1}$), the calculated vector $\chi_{k-1}$ that contains 2n+1 sigma points is given as

(6) $$\begin{array}{l} P_{k-1}=D_{k-1}D_{k-1}^{T}\\ \chi_{k-1}={\left[\begin{array}{c} {\hat{x}}_{k-1}\\ {\hat{x}}_{k-1}+\sqrt{n+\lambda }\times D_{k-1}\\ {\hat{x}}_{k-1}-\sqrt{n+\lambda }\times D_{k-1} \end{array}\right]}^{T} \end{array}$$

where $D_{k-1}$ is the lower triangle matrix in the above Cholesky decomposition for $P_{k-1}$; $\lambda =\alpha^{2}(n+\kappa )-n$ is the scale factor, α and κ are constants, α determines the spread of the sigma points around ${\hat{x}}_{k-1}$ (usually set as 0 or 3−n), and κ is a constant (usually set as 0). The corresponding weights of the produced sigma points are given as:

(7) $$\begin{array}{l} W_{0}^{n}=\lambda /(n+\lambda )\\ W_{0}^{c}=\lambda /(n+\lambda )+(1-\alpha^{2}+\beta )\\ W_{i}^{n}=W_{i}^{c}=1/2(n+\lambda ),i=1,2,\cdots ,2n \end{array}$$

where $W_{i}^{n}$ denotes weight of mean value and $W_{i}^{c}$ is weight of the covariance for the i-th sigma point; β is used to fuse prior information of random variables, and β= 2 is generally utilized for the Gauss distribution.

Step 3: Update the state.

(8) $$\begin{array}{l} \chi_{k|k-1}=f(\chi_{k-1})\\ {\hat{x}}_{k|k-1}=\sum_{i=0}^{2n}W_{i}^{n}\chi_{i,k|k-1}\\ P_{k|k-1}=\sum_{i=0}^{2n}W_{i}^{c}(\chi_{i,k|k-1}-{\hat{x}}_{k|k-1})\times (\chi_{i,k|k-1}-{\hat{x}}_{k|k-1}){}^{T}+Q_{k} \end{array}$$

where ${\hat{x}}_{k|k-1}$ is the updated mean value of the state, and $P_{k|k-1}$ denotes the corresponding covariance of ${\hat{x}}_{k|k-1}$.

Step 4: Update the measurement.

(9) $$\begin{array}{l} y_{k|k-1}=h(\chi_{k|k-1})\\ {\hat{y}}_{k}=\sum_{i=0}^{2n}W_{i}^{n}y_{i,k|k-1}\\ P_{yy}=\sum_{i=0}^{2n}W_{i}^{c}(y_{i,k|k-1}-{\hat{y}}_{k})\times (y_{i,k|k-1}-{\hat{y}}_{k}){}^{T}+R_{k} \end{array}$$

where ${\hat{y}}_{k}$ is the updated mean value of the predicted measurement, and $P_{yy}$ denotes the corresponding covariance of ${\hat{y}}_{k}$.

Step 5: Update the SUKF filter

(10) $$\begin{array}{l} P_{xy}=\sum_{i=0}^{2n}W_{i}^{c}(\chi_{i,k|k-1}-{\hat{x}}_{k|k-1})\times (y_{i,k|k-1}-{\hat{y}}_{k}){}^{T}\\ K_{k}=P_{xy}P_{yy}^{-1}\\ {\hat{x}}_{k}={\hat{x}}_{k|k-1}+K_{k}(y_{k}-{\hat{y}}_{k})\\ P_{k}=P_{k|k-1}-K_{k}P_{yy}K_{k}^{T} \end{array}$$

where $K_{k}$ is the gain matrix; ${\hat{x}}_{k}$ is the updated SUKF-based state parameter, and $P_{k}$ is the corresponding covariance matrix of ${\hat{x}}_{k}$.

Step 6: Calculate the posterior observation residual

In Step 5, we can obtain the estimated parameter dx (namely, ${\hat{x}}_{k}$ above), then we can calculate the posterior observation residual v and corresponding VC matrix $C_{vv}$ by

(11) $$\begin{array}{l} v=Bdx-l\\ C_{vv}=C_{l}-B(B^{T}C_{l}^{-1}B){}^{-1}B^{T} \end{array}$$

where B is the design matrix; in this study, $B=\left[\begin{array}{cc} \Gamma & \Lambda \\ \Gamma & 0 \end{array}\right]$ as in Equation (2); l is a mis-closure vector of the DD PL observation, and $C_{l}$ is the VC matrix of l.

Step 7: Calculate the standardized residual

The standardized residual for code and phase observations on the basis of v and $C_{vv}$ in Equation (11) can be described as

(12) $${\bar{v}}_{k}=\frac{v_{k}}{\sqrt{C_{vv}(k,k)}}$$

where $v_{k}$ denotes the posterior observation residual of the k-th observation, $C_{vv}(k,k)$ is a scalar denoting the k-th diagonal element of $C_{vv}$. 

Step 8: Calculate the error discriminant statistics

The constructed error discriminant statistics ($\Delta {\bar{v}}_{k}$) on the basis of ${\bar{v}}_{k}$ can be described as

(13) $$\Delta {\bar{v}}_{k}=\frac{{\bar{v}}_{k}}{\sum_{j=1,j\neq k}^{n}\left|{\bar{v}}_{j}\right|/(n-1)}$$

Step 9: Calculate the robust factor 

The calculated robust factor is used for adjusting the equivalent weight matrix. In this study, we adopt the IGGIII scheme. The three-segment function can be described as

(14) $$\lambda_{k}=\{\begin{array}{ccc} 1 & & \left|\Delta {\bar{v}}_{k}\right|\leq k_{0}\\ \frac{\left|\Delta {\bar{v}}_{k}\right|{\left(k_{1}-k_{0}\right)}^{2}}{k_{0}{\left(k_{1}-\left|\Delta {\bar{v}}_{k}\right|\right)}^{2}} & & k_{0}<\left|\Delta {\bar{v}}_{k}\right|\leq k_{1}\\ \infty & & \left|\Delta {\bar{v}}_{k}\right|>k_{1} \end{array}$$

where $k_{0}$ and $k_{1}$ are constants, the reasonable ranges are generally 1.0 $\leq k_{0}\leq$ 2.0 and 2.5 $\leq k_{0}\leq$ 8.0.

Step 10: Update the RUKF filter 

We update the UKF filter solution by using the abovementioned robust factor.

(15) $$P_{yy}=\sum_{i=0}^{2n}W_{i}^{c}(y_{i,k|k-1}-{\hat{y}}_{k})\times {\left(y_{i,k|k-1}-{\hat{y}}_{k}\right)}^{T}+\lambda_{k}\cdot R_{k}$$

where the meaning of $P_{yy}$ is the same as the expression in Equations (9) and (10). The subsequent filter updating is the same as Step 5. 

### 2.3. PAR for PL Positioning 

In the conventional indoor PL RTK processing, all PL DD ambiguities will be involved in the PL-AR using the LAMBDA method. The RUKF can improve the accuracy of the float solution to some extent, but the whole set of PL DD ambiguity cannot be fixed successfully. Thus, a PAR strategy for PL RTK positioning is introduced.

After conducting the RUKF, the parameters of the baseline vector and ambiguity float solution in Equation (2) denote $\hat{a}$ and $\hat{b}$, and the corresponding VC matrix is composed of $Q_{\hat{a}}$ and $Q_{\hat{b}}$. The subset of the PL DD ambiguities can be described as

(16) $$\hat{b}=\left[\begin{array}{c} {\hat{b}}_{1}\\ {\hat{b}}_{2} \end{array}\right],Q_{\hat{b}}=\left[\begin{array}{cc} Q_{{\hat{b}}_{1}} & Q_{{\hat{b}}_{1}{\hat{b}}_{2}}\\ Q_{{}_{{\hat{b}}_{2}{\hat{b}}_{1}}} & Q_{{\hat{b}}_{2}} \end{array}\right]$$

where $\hat{b}$ is the entire set of estimated float ambiguity solutions; ${\hat{b}}_{1}$ is the optimal subset, which can be easily fixed using the LAMBDA method; and ${\hat{b}}_{2}$ is the remaining subset, which is difficult to fix due to certain errors in the corresponding DD phase observation.

The selecting criterion ${\hat{b}}_{1}$ is the core of the PAR for PL. In general, the SNR, elevation, posterior observation residual, the estimated variance information of float ambiguity, and the bootstrapping AR success rate or ratio value when conducting LAMBDA can be adopted to the identify the criterion of the optimal subset. In this study, we use the error discriminant statistics ($\Delta \bar{v}$) in Equation (13) to select subset ${\hat{b}}_{1}$. 

We sort $\Delta \bar{v}$ of all PLs in ascending order, which can be described as

(17) $$\Delta \bar{V}=\left\{\Delta {\bar{v}}_{1},\Delta {\bar{v}}_{2},\cdots \Delta {\bar{v}}_{n}|\Delta {\bar{v}}_{1}<\Delta {\bar{v}}_{2}<\cdots <\Delta {\bar{v}}_{n}\right\}$$

where $\Delta {\bar{v}}_{k}$ represents the elevation in k-th order. If the number of the subset ${\hat{b}}_{1}$ (m) is pre-given, then the program can easily obtain the subset ${\hat{b}}_{1}$.

(18) $${\hat{b}}_{1}=\left\{N_{1},N_{2},\cdots N_{m}|\Delta {\bar{v}}_{1}<\Delta {\bar{v}}_{2}<\cdots <\Delta {\bar{v}}_{m}\right\}$$

where N denotes the PL DD ambiguity float solution. If the subset ${\hat{b}}_{1}$ has been successfully fixed using LAMBDA, then the fixed solution denotes ${\overset{⌣}{b}}_{1}$. Accordingly, we can update the remaining subset ${\hat{b}}_{2}$, which can be described as

(19) $$\begin{array}{l} {\overset{⌣}{b}}_{2}={\hat{b}}_{2}-Q_{{\hat{b}}_{2}{\hat{b}}_{1}}Q_{{\hat{b}}_{1}}^{-1}({\hat{b}}_{1}-{\overset{⌣}{b}}_{1})\\ Q_{{\overset{⌣}{b}}_{2}}=Q_{{\hat{b}}_{2}}-Q_{{\hat{b}}_{2}{\hat{b}}_{1}}Q_{{\hat{b}}_{1}}^{-1}Q_{\hat{b}\hat{a}} \end{array}$$

If the subset ${\hat{b}}_{1}$ cannot be successfully fixed, then we continue to decrease the number of the subset ${\hat{b}}_{1}$ (m) and repeat the above steps until m is less than 3. We then exit the program of PAR.

### 2.4. Data Processing

**Figure 1:**
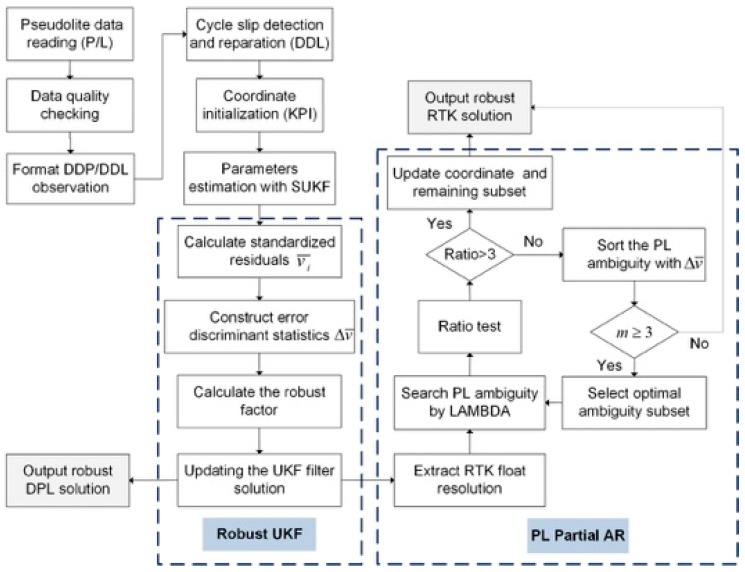
Flowchart of indoor PL positioning based on our proposed algorithm.

In this study, we propose a reliable indoor PL positioning method based on the RUKF and PAR. The relative positioning model and KPI method are adopted. If only code observation is used for positioning, we call it the DPL model, while if the high-precision carrier phase is used, we call it the RTK model. For the single-frequency PL system, the polynomial fitting method is used for cycle slip detection and reparation for carrier phase observations. The initial coordinate value is pre-given within a relatively accurate range when KPI is conducted. The popular LAMBDA method is used for PL-AR. Figure 1 shows the flowchart of our proposed method.

## 3. Experimental Results and Analysis

### 3.1. Observation Platform of the Indoor Positioning System

**Figure 2:**
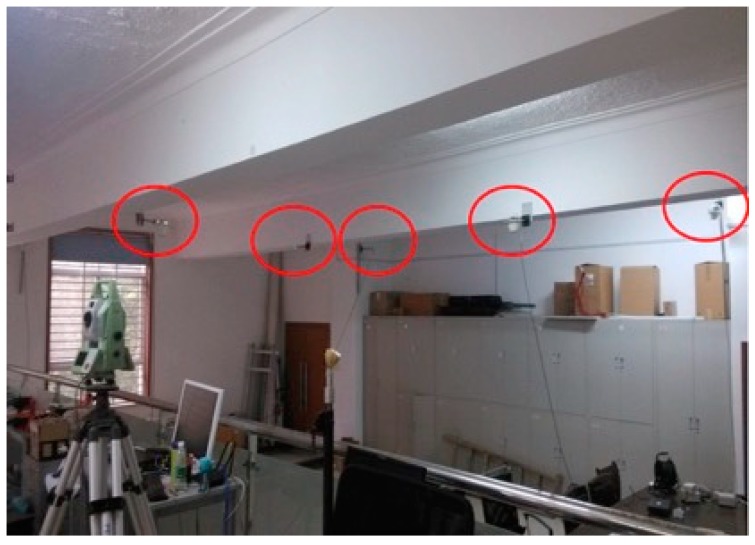
Scene of the indoor PL distribution.

The PL positioning system is set up in a 10 m × 7 m × 4 m laboratory with five PLs mounted on the ceiling. The model of PL instrument is GSG-L1, which can generate an L1 carrier that is BPSK modulated with the C/A code and navigation signal. In this local coordinate, the origin point is located at the center of the laboratory. The coordinates of PLs were accurately determined in advance by a total station. As shown in Figure 2, the locations of PLs are indicated by red circles.

**Figure 3:**
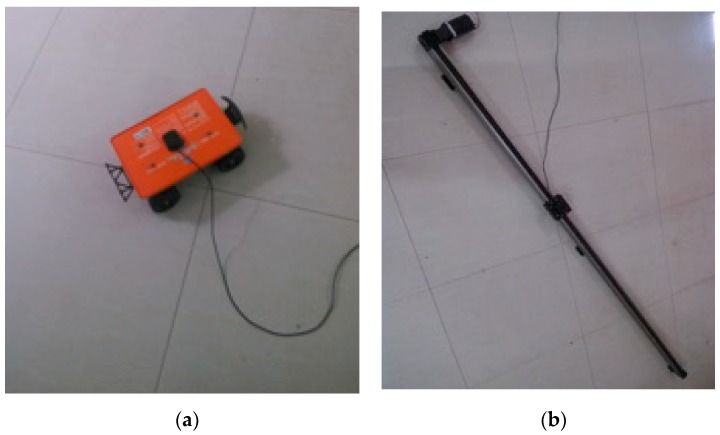
Experimental data acquisition scene. (**a**): a mobile car is remotely controlled for free movement; (**b**): a rover station is moving straight along the fixed rail.

The base and rover stations use Universal Software Radio Peripheral (USRP) as the frontend of the software receiver. The main function of USRP is to mix the L1 radio frequency signal to an intermediate frequency signal, and then complete the digitalization process. The following acquisition and tracking processes are all done by a self-developed software receiver, which brings great convenience to the research of indoor PL positioning systems. Figure 3 shows the experimental data acquisition scene. Each grid on the floor is a square of 0.6 m × 0.6 m. The base station is fixed on known points, and the rover station can move arbitrarily on a mobile car or along fixed rail. 

### 3.2. DPL Model

**Figure 4:**
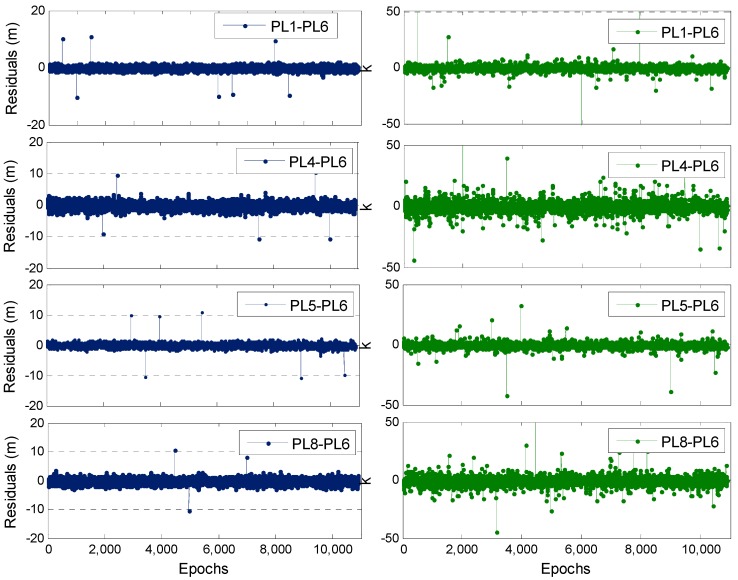
Observation posterior residuals and error discriminant statistics (k) of the four PL DD code observations.

In this test, a static short baseline is used, and the observation conditions are relatively good. A total of approximately 11,000 observation epochs are used. Five PLs are synchronously observed during the entire observation period; thus, four DD code observations can be formed, we denote them as PL1-PL6, PL4-PL6, PL5-PL6 and PL8-PL6. The data processing method in Section 2.4 is used to conduct static robust DPL positioning for this test. The initial coordinates of UKF are provided accurately in the range of 0.1 m. A large gross error of 10 m is randomly added to some DD code observations on approximately 20 epochs to verify the resistance of the proposed algorithm to the gross errors of code observations and the small gross errors of the original observation data. Figure 4 shows the posterior residuals of four DD code observations and the corresponding sequence of error discriminant statistics ($\Delta {\bar{v}}_{i}$, denotes k as well) in Equation (13) during the entire observation period.

The observation posterior residuals can clearly reflect the anomalies of four PL DD code observations, especially large gross errors. A comparison of the error discriminant statistics (k) indicates that the corresponding values of k for some epochs with the 10 m gross error increase dramatically compared with those for other normal observation epochs. Apart from the epochs with artificial gross errors, many other epochs also show evident anomalies. This finding indicates that the error discrimination statistics can identify some small gross errors. In this experiment, $k_{0}$ and $k_{1}$ in Equation (14) are set to 2 and 8, respectively, when conducting the RUKF for indoor PL positioning. According to the absolute value of k, the observation environment can be divided into three situations as follows. In Situation #1, no abnormality is found in the observations, and the corresponding equivalent weight matrix does not need to be adjusted. In Situation #2, a certain small gross error exists in the observations, and the corresponding equivalent weight matrix needs weight reduction processing. In Situation #3, some large gross errors exist in the observatons, and the corresponding equivalent weight matrix needs weight abandonment processing. The specific method is presented in Equation (14).

Table 1 shows the epoch number statistics of various DD PLs under the three situations mentioned above. The statistical results in Situation #1 indicate that most of the single DD code observations are normal without anomaly, but the epoch number of all the DD code observations that are simultaneously normal is relatively few at only 3694. The statistical results in Situation #2 show that most epochs, which account for approximately 63% of the total epochs, are affected by a minor gross error to varying extent. The statistical results in Situation #3 reveal that more than 300 epochs with a gross error of 10 m have relatively large gross errors in the original code observation.

**Figure 5:**
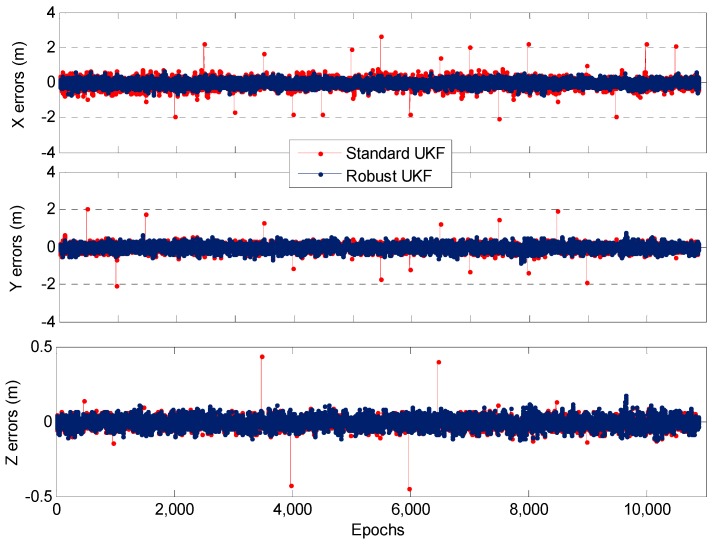
Comparison of SUKF-/RUKF-based DPL positioning errors in X/Y/Z directions for one static test.

Figure 5 shows the positioning error sequence for SUKF and RUKF. The SUKF lacks resistance to observation anomalies, and its positioning results are worse than those of RUKF when gross errors exist for many epochs, especially large gross errors. This disadvantage of SUKF is evident. By contrast, the proposed RUKF algorithm can weaken the adverse effect of observation anomalies and improve the positioning accuracy.

**Figure 6:**
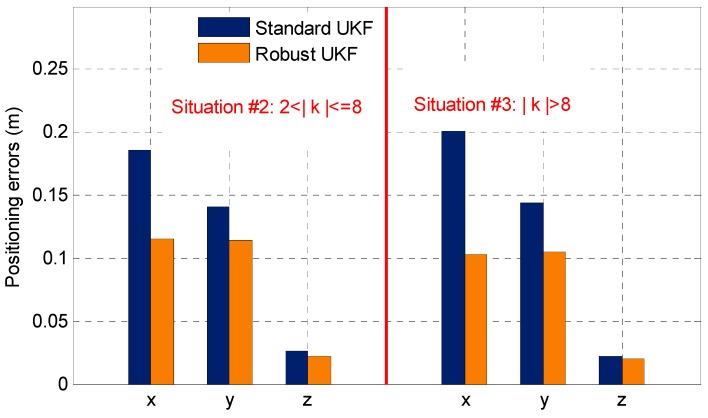
Statistical results of the SUKF-/RUKF-based DPL average positioning errors in Situations #2 and #3 for one static test.

Figure 6 shows the DPL average positioning error statistics (computed by the absolute value of every positioning errors) of SUKF/RUKF in Situations #2 and #3. A comparison of the statistical results shows that the RUKF performs better in Situations #2 and #3 than the SUKF, especially when large gross errors exist such as in Situation #3. In the case of small gross errors (Situation #2), RUKF has a decimeter-level improvement for the indoor PL positioning accuracy compared with SUKF because the RUKF uses a robust factor to adjust the equivalent weight matrix automatically. The RUKF also conducts weight reduction processing for anomalous observations, which can adaptively weaken its negative effect to some extent.

### 3.3. RTK Model

The accuracy and stability of the DD carrier phase observation show much better performance than those of the DD code observation. The former is also less susceptible to interference and has smaller multipath effects under the indoor environment than the latter. However, if no robust strategies are applied under the occurrence of abnormal interference, then the final positioning accuracy and reliability will be affected; in particular, the PL-AR will be challenging. This phenomenon is evident, especially for indoor kinematic positioning. In this study, the superiority of the proposed RUKF combined with the PAR algorithm is verified by static and kinematic tests in two cases. One case is that the PL ambiguity can be easily solved, and the other is that the the observation exists some abnormalities and PL ambiguity cannot be solved successfully using the conventional PL-AR method.

#### 3.3.1. Static Test

**Figure 7:**
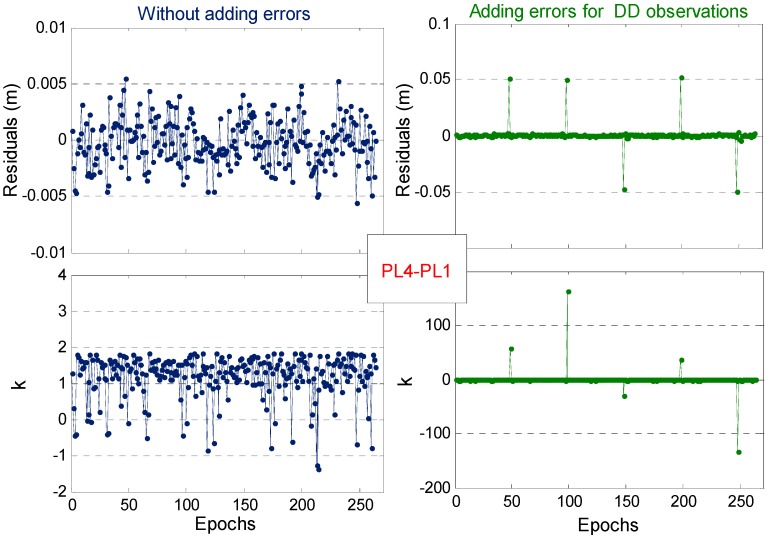
Posterior observation residuals and error discriminant statistics (k ) of the one PL DD carrier phase observation. Left: Original PL4-PL1 observations without artificial errors. Right: PL4-PL1 observations with artificial errors of 5 cm on some epochs.

In this experiment, one static short baseline is used, and the observation conditions are relatively good. A total of 270 observation epochs are used. Five PLs are observed synchronously during the entire observation period. Each epoch can form four carrier phase DD observations, denoted as PL4-PL1, PL5-PL1, PL6-PL1 and PL8-PL1. The PL4-PL1 observation for some epochs is added with a gross error of 5 cm without affecting the normal PL-AR to verify that the RUKF can identify and resist a certain small gross error for carrier phase observation. Figure 7 shows the posterior residuals and the corresponding error discrimination statistics (k) of the original PL4-PL1 observations, and the corresponding results after artificially adding a gross error of 5 cm. In Figure 7, the original carrier phase observations are relatively stable, most of the posterior residuals are less than 5 mm, and the corresponding k values are less than 2. Therefore, all epoch observations are not abnormal. After a small gross error of 5 cm is added, the corresponding epoch and posterior residual also exhibit a large jump. The corresponding k values are highly sensitive to detect the occurrence of the anomaly.

**Figure 8:**
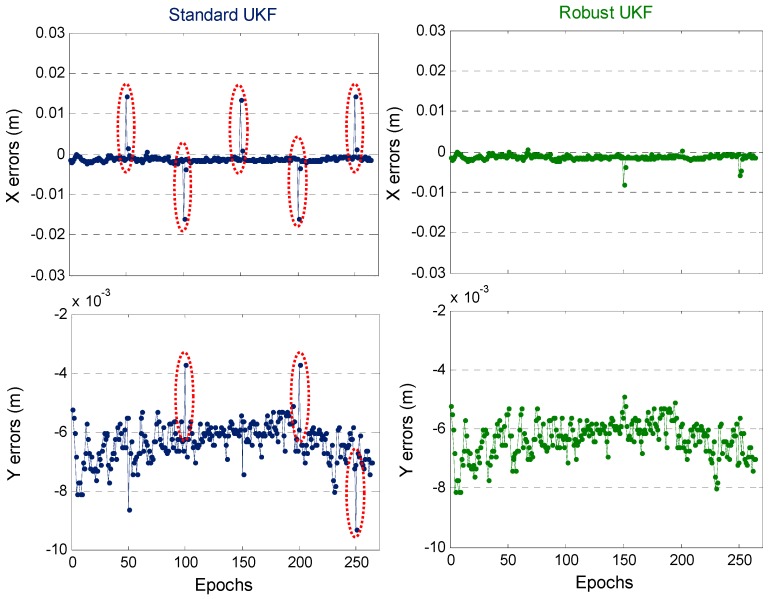
Comparison of SUKF-/RUKF-based RTK positioning errors for one static test.

When conducting KPI for the short baseline, the ambiguity is easily fixed as long as the given initial coordinates have high accuracy. The fix and hold mode is used to gain the ambiguity solution between successive epochs, and the coordinates for all epochs are fixed solutions in this test. Figure 8 shows the sequence of positioning errors for SUKF and RUKF. The SUKF-/RUKF-based positioning results have no differences in the epochs without gross error. However, for the epochs with a gross error of 5 cm (within the red ellipse in Figure 8), a centimeter-level jump occurs in the SUKF-based positioning results. By contrast, the RUKF weakens the influence of gross errors and does not show large abnormality in its positioning results.

The SUKS-based indoor PL-AR usually fails when gross errors exist in carrier phase observation. A gross error of 0.15 m is artificially added to the PL4-PL1 observations on the 100th epoch to verify the validity of our proposed RUKF and PAR strategy for indoor PL RTK positioning for the abovementioned static short baseline data. Three experimental schemes are adopted for data processing: Scheme #1 based on SUKF, Scheme #2 based on RUKF, and Scheme # 3 based on RUKF combined with the PAR algorithm. 

**Figure 9:**
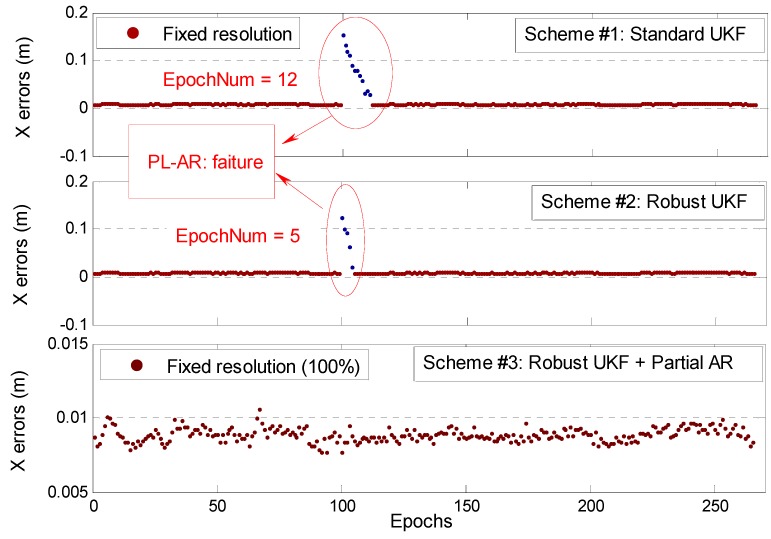
Comparison of the RTK positioning errors in the case of three different experimental schemes for one static test.

Figure 9 shows the positioning results for the three experimental schemes. For the 100th epoch, the SUKF-based PL-AR fails and has a jump of the positioning result of approximately 0.16 m due to the lack of resistance to abnormal observations. The RUKF cannot achieve the ambiguity fixed solutions as well, and shows a jump of the positioning result of approximately 0.12 m. Further comparison shows that SUKF needs 12 epochs to achieve ambiguity re-fixing, whereas RUKF needs five epochs. The reason is that the RUKF cannot realize PL-AR for the 100th epoch but still weakens the effect of adding gross errors to a certain extent. The accuracy of float ambiguity solution of RUKF is higher than that of SUKF and thus has a relatively faster filtering convergence speed for subsequent epochs. Scheme #3 adopts a PAR strategy based on RUKF. The PL-AR procedure does not consider all ambiguities for searching together, and an ambiguity subset that contains three other normal observations is selected for partial PL-AR. Therefore, the approach can achieve fixed solutions for all epochs and improves positioning accuracy.

#### 3.3.2. Kinematic Test 

In this test, a fixed rail is used, and a mobile car with a PL receiver antenna travels from one end to the other. A total of 270 observation epochs are used. Five PLs are observed synchronously during the entire observation period. Each epoch can form four carrier phase DD observations. The advantage of our proposed method is verified in the case of successful and failed PL-AR with conventional methods.

**Figure 10:**
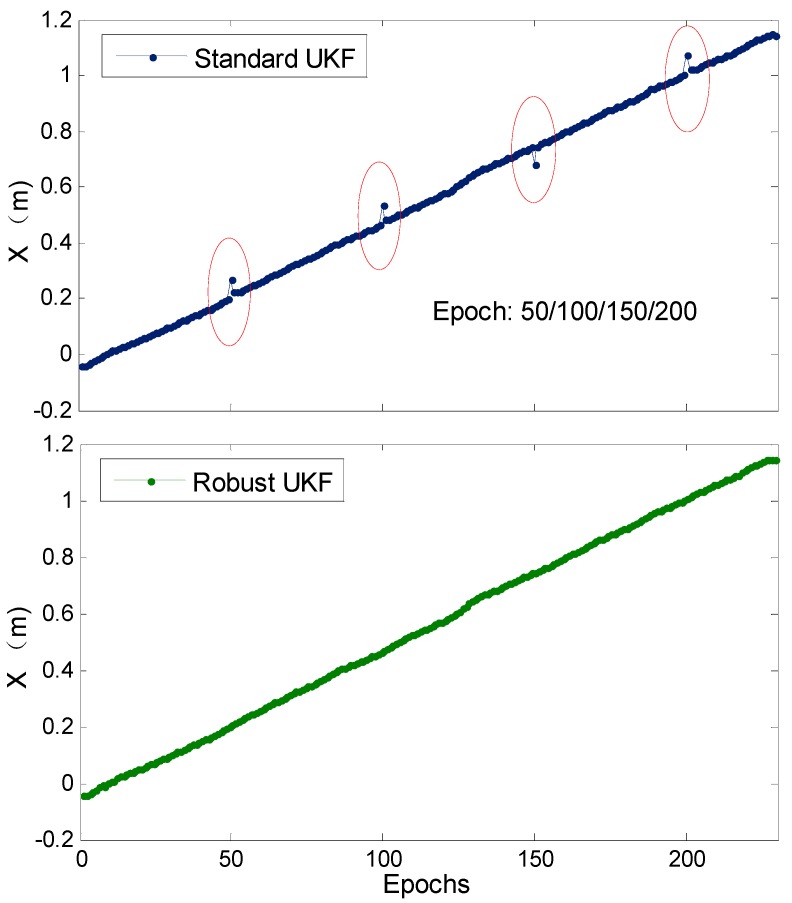
Comparison of SUKF-/RUKF-based RTK positioning errors (X-direction) in the case of adding certain gross errors with 0.1 m for some epochs for the kinematic rail test.

A gross error of 0.1 m is added to one carrier phase observation for some epochs, and the SUKF and RUKF are used for kinematic positioning. The initial coordinates given for the first epoch are relatively accurate, the fix and hold mode is adopted to gain the ambiguity solution between successive epochs, and the coordinates of all epochs are fixed solutions in this experiment. Figure 10 shows the positioning results of two different schemes. The SUKF-based X-directional positioning results show several centimeter-level jumps for the epochs with gross errors (within the red ellipse in Figure 10), whereas RUKF can effectively weaken the effect of adding gross errors and has no large fluctuation in its positioning results.

**Figure 11:**
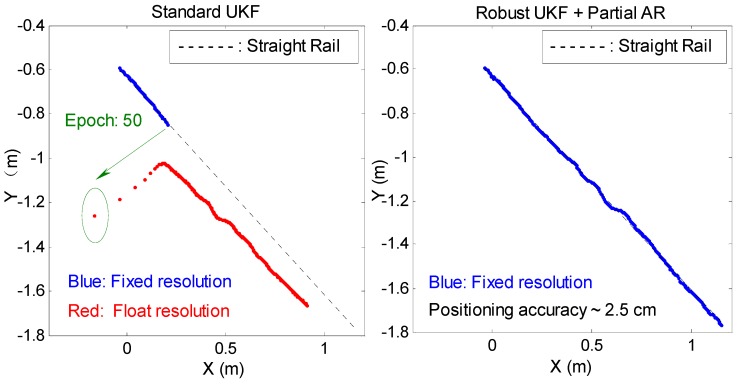
Planar trajectory derived from the RTK positioning results of two different experimental schemes for kinematic rail test.

In kinematic positioning of a moving rover station, the initial coordinate value of the current epoch is usually based on the coordinates of the previous epoch. A large deviation in the positioning result of one epoch negatively affects the subsequent initialization of coordinates and thus leads to filtering divergence and positioning failure. Therefore, the proposed RUKF and PAR algorithm is more necessary than the static positioning. In this experiment, a gross error of 0.15 m is added to one carrier phase observation for the 50th epoch. Two different data processing schemes, namely, SUKF and RUKF and PAR, are adopted for kinematic positioning. The single-epoch PL-AR model is used during the successive epochs. Figure 11 shows the planar trajectory derived from the kinematic positioning results of the two different schemes. For the 50th epoch, the SUKF-based PL-AR fails and has a deviation of several decimeters for the positioning result, which is transmitted to the subsequent initialization of coordinates. The subsequent filtering of epochs cannot converge and continues to diverge along with the corresponding positioning results due to the large deviation. Figure 11 shows that the plane trajectory correspondingly deviates from the true straight rail. When the RUKF and PAR algorithm is adopted, the effect of adding the gross error is weakened to a certain extent, and the ambiguity subset containing three other normal observations is selected and successfully realizes the partial PL-AR. The positioning accuracy based on our proposed method is better by 2–3 cm than the planar positioning trajectory and the true straight rail.

## 4. Conclusions

Our observations in this study are prone to abnormalities due to the complex environment of indoor PL positioning. The traditional parameter estimation method lacks the effective ability of anomaly recognition and anti-interference, and the conventional PL-AR becomes challenging. This study presents a reliable indoor PL positioning method based on the RUKF and PAR strategy. The error discriminant statistic is constructed using posterior residuals of the observations, and the equivalent weight matrix of observation is adaptively adjusted by the calculated robust factor. The optimal ambiguity subset is selected to conduct the partial PL-AR on the basis of the error discriminant statistics. The superiority of the proposed algorithm for indoor code-based DPL and phase-based RTK positioning is verified using static and kinematic observation data. Some valuable conclusions are obtained as follows.

1. Compared with the SUKF algorithm, the RUKF algorithm can effectively weaken the anomalous effect of PL code observations and improve the accuracy and reliability of indoor DPL positioning, especially when certain large gross errors exist.
2. RUKF can identify small gross errors (centimeter-level) of PL carrier observations and achieve the corresponding indoor RTK positioning accuracy of fixed solutions at a centimeter-level improvement.
3. Compared with SUKF, RUKF can improve the accuracy of ambiguity float solution and the re-convergence speed when the carrier phase observation has relatively large gross errors. However, RUKF cannot achieve PL-AR successfully. The proposed RUKF combined with PAR strategy can achieve partial PL-AR for the selected ambiguity subset and obtain an accurate fixed solution. The advantages of our proposed algorithm are important for indoor PL kinematic positioning.

Currently, most of the PL systems are still only capable of high-positioning adoption of the KPI, which brings some inconveniences in application. In our future work, we will form a combination for indoor PL and ultra-wideband (UWB) positioning, and it is expect that the indoor UWB-assisted PL positioning can avoid the disadvantage of KPI and have a better positioning performance.

## Figures and Tables

**Table 1:** Epoch number statistics of various DD PLs in three different situations.

	Situation #1	Situation #2	Situation #3
PL1-PL6	9887	949	29
PL4-PL6	7846	2819	200
PL5-PL6	10014	826	25
PL8-PL6	8255	2486	124
All DD PLs	3693	6794	378
